# Development of educational videos about bathing in bed newborns admitted to a neonatal unit

**DOI:** 10.1590/0034-7167-2022-0778

**Published:** 2023-11-13

**Authors:** Maria Paula Custódio Silva, Cinthia Lorena Silva Barbosa Teixeira, Juliana da Silva Garcia Nascimento, Kleiton Gonçalves do Nascimento, Rui Carlos Negrão Baptista, Luciana Mara Monti Fonseca, Divanice Contim

**Affiliations:** I Universidade Federal Triângulo Mineira. Uberaba, Minas Gerais, Brazil; II Escola Superior de Enfermagem de Coimbra. Coimbra, Portugal; III Universidade de São Paulo. Ribeirão Preto, São Paulo, Brazil

**Keywords:** Nursing Care, Health Education, Newborn, Baths, Instructional Film and Video, Atención de Enfermería, Educación en Salud, Recién Nacido, Baños, Película y Video Educativos, Cuidados de Enfermagem, Educação em Saúde, Recém-Nascido, Banhos, Filmes e Vídeo Educativo

## Abstract

**Objective:**

to develop and analyze evidence of content validity of educational videos about bathing newborns in bed in a neonatal unit.

**Method:**

applied and methodological research, carried out from December/2020 to February/2022, in three phases: pre-production, production, post-production. Validity was carried out by nurses specializing in social communication and nursing professionals, including the Brazilian Sign Language and assessment by nursing students. The Content Validity Index and Cronbach’s alpha above 0.8 were considered for analysis.

**Results:**

the videos were entitled “Best practices: bathing newborns in the heated crib” and “Best practices: bathing newborns in the incubator”, lasting seven minutes each, divided into six scenes that demonstrated the approach to parents, environment and material organization, preparing newborns, bathing and after-bath care.

**Conclusion:**

the videos will support permanent education processes, academic training and professional training in nursing.

## RESUMO

**Objetivo:**

desenvolver e analisar evidências de validade de conteúdo de vídeos educativos sobre o banho do recém-nascido no leito em unidade neonatal.

**Método:**

pesquisa aplicada e metodológica, desenvolvida de dezembro/2020 a fevereiro/2022, em três fases: pré-produção, produção, pós-produção. A validação foi realizada por enfermeiros doutores especialistas na área da comunicação social e profissionais da enfermagem, incluindo-se a Língua Brasileira de Sinais e avaliação por estudantes de enfermagem. O Índice de Validade de Conteúdo e do alfa de Cronbach acima de 0,8 foram considerados para análise.

**Resultados:**

os vídeos intitularam-se “Boas práticas: banho do recém-nascido no berço aquecido” e “Boas práticas: banho do recém-nascido na incubadora”, com duração de sete minutos cada, dividido em seis cenas que demonstraram abordagem dos pais, organização do ambiente e materiais, preparo do recém-nascido, banho e cuidados após o banho.

**Conclusão:**

os vídeos subsidiarão processos de educação permanente, formação acadêmica e capacitação profissional em enfermagem.

## RESUMEN

**Objetivo:**

desarrollar y analizar evidencias de validez de contenido de videos educativos sobre el baño de recién nacidos en la cama en una unidad neonatal.

**Método:**

investigación aplicada y metodológica, realizada de diciembre/2020 a febrero/2022, en tres fases: preproducción, producción, postproducción. La validación fue realizada por enfermeros especialistas en el área de comunicación social y profesionales de enfermería, incluyendo la Lengua de Signos Brasileña y evaluación por estudiantes de enfermería. Se consideró para el análisis el Índice de Validez de Contenido y el alfa de Cronbach superior a 0,8.

**Resultados:**

los videos se titularon “Buenas prácticas: bañar al recién nacido en la cuna caliente” y “Buenas prácticas: bañar al recién nacido en la incubadora”, con una duración de siete minutos cada una, dividida en seis escenas que mostraban el acercamiento de los padres, organización del ambiente y materiales, preparación del recién nacido, baño y cuidados posteriores al baño.

**Conclusión:**

los videos apoyarán procesos de educación permanente, formación académica y formación profesional en enfermería.

## INTRODUCTION

The way to perform daily hygiene care for newborns (NB) hospitalized in neonatal units depends on gestational age (GA), weight, clinical condition and presence of devices. Ocular, oral and intimate hygiene are part of this care. For other parts of the body, an interval of 96 hours or more is considered, in order to avoid skin infections, temperature variation and stress^(1, 2)^.

Immersion bath is indicated for those NB who have a GA of over 36 weeks, clinical stability and absence of devices such as peripheral or central venous access, drains, oral and nasotracheal tube and non-invasive ventilation^(3)^. When NB do not fit these criteria, the bath should be performed in the bed^(3)^. This procedure has been differing in hospital institutions, mainly with regard to intervals and necessary care^(3)^, such as winding, handling in pairs, heat source turned on, adequate room temperature and shorter execution time^(4)^.

Bearing in mind the importance of paying greater attention to NB’s care needs, especially in neonatal units and at times of excessive handling, such as bathing, basing nursing care on more robust scientific findings capable of directing the best practices that encourage the safety of this clientele, it becomes necessary to adopt attractive training strategies, with a view to acquiring knowledge and behavioral changes in students and nursing professionals^(5)^.

Despite the lack of irrefutable scientific evidence regarding the effectiveness of using pedagogical videos for teaching and learning in nursing, especially in the context of neonatal care, this technology has been indicated for the development of clinical skills and increased self-confidence and self-efficacy, given its contemporaneity, practicality and ability to motivate learners^(5)^.

It is also believed that the educational video is capable of improving the understanding of the care provided by nursing and aligning the assistance provided in this context, from academic training to work practice, with facilitated access, nowadays, via cell phone, which allows the deliberate visualization of procedures before experiencing a simulation or real care practice^(6, 7)^. Also, it strives to minimize aggravating situations for safety, such as exposure to cold, stress, excessive handling and preservation of devices^(3)^.

Given the importance of the theme and the absence of studies that produced audiovisual learning objects, based on the best evidence for bathing the NB’s bed in an intensive care unit, the questions arose: Are the educational videos about bathing NB in bed valid in terms of content and appearance to be used as an educational health technology for nursing staff professionals and nursing students? Is the inclusion of Brazilian Sign Language (LIBRAS) in educational videos in line with current regulations?

## OBJECTIVE

To develop and analyze evidence of content validity of educational videos about bathing NB in bed in a neonatal unit.

## METHODS

### Ethical aspects

The study was conducted in accordance with national and international ethics guidelines, and was approved by the Research Ethics Committee of the *Universidade Federal do Triângulo Mineiro*, whose opinion is attached to this submission. The online Informed Consent Form was obtained from all individuals involved in the study.

### Study design, period, and place

This is applied research and methodological production and technological validity, guided by the framework of quality improvement studies-SQUIRE of the EQUATOR network, developed from December 2020 to February 2022 in three phases, pre-production, production and post-production^(8)^, visualized in Chart 1 below.

**Chart 1:** Process of production and validity of educational videos about bathing in the bed of newborns hospitalized in neonatal units considering the pre-production, production and post-production phases, Uberaba, Minas Gerais, Brazil, 2022

**Pre-production**	Elaboration of script and storyboard, production team recruitment, raising of physical, financial, technological and human resources;	
	Script structure:	Target audience, objectives, setting, scenes/stages, dummy, team, equipment and materials.
	Storyboard structure for recording:	Format of a comic book arranged in columns, containing the descriptions of scenes in drawings and shooting angles.
	Storyboard structure for editing:	Texts and audio aspects such as narration and background music.
**Production**	Script and storyboard content and appearance validity with specialists from December 2020 to May 2021, rehearsal with actors, recording of scenes, development of images, animations and narration/audio recording.	
	Script and storyboard validity criteria:	Objective, content, relevance, environment, verbal language and inclusion of topics^(9)^.
	Production team:	Researchers, one PhD and two PhD students, all with professional experience in neonatology and audiovisual technicians with experience in video recording and editing.
	Recording environment:	Neonatal Intensive Care Unit.
**Post-production**	Composition of all elements of the storyboard in a continuous sequence of scenes with the inclusion of texts. Afterwards, it underwent a second validity stage that took place during the months of December to February 2022.	
	Validity and assessment criteria of the edited video:	Functionality, usability, efficiency, audiovisual technique, environment and procedure^(9)^.

### Population or sample; inclusion and exclusion criteria

Participants in script and storyboard validity were 16 PhD nurses, selected according to the inclusion criteria proposed by Fering (1987)^(10)^, adapted and verified through the Brazilian National Council for Scientific and Technological Development (*Conselho Nacional de Desenvolvimento Científico e Tecnológico*) platform, considering up: master’s degree in nursing (4 points); master’s degree in nursing with dissertation in the area of interest of the study (1 point); doctoral thesis in the study area (2 points); clinical experience of at least one year in the area of interest (1 point); certificate of clinical practice (specialization) in the area of interest of the study (2 points); publication relevant to the area of interest (2 points); and publication of an article on the subject in a reference journal (2 points). For nurses to be selected, they must obtain a minimum of five points, among those who had a PhD.

Sixteen PhD nursing from the first stage, three specialists in the area of social communication and 43 nursing team members as well as 23 nursing students as evaluators, participated in the validity of the edited video.

To be selected as specialists in social communication, professionals should have a degree in social communication, experience with technical support, programming or networking and experience with video editing. To be selected as members of nursing team, they should be working in the maternal and child area for more than five years.

For nursing students to be selected as evaluators, they should be enrolled in a higher education nursing course and have taken courses with content on pediatric nursing, gynecology and obstetrics nursing, and women’s, adolescent, and child health nursing. Selection and recruitment were carried out using the snowball technique, which consisted of nominating participants by themselves successively^(11)^.

LIBRAS validity was performed by three specialists in LIBRAS, selected and recruited through the snowball technique, according to the criteria: acting as a LIBRAS professor or being an interpreter in LIBRAS for more than two years.

### Study protocol

For a better description and understanding of the development process of the proposed pedagogical tools, in the first phase, pre-production, an integrative literature review was carried out a priori to list the stages of NB bed bath to be included in the script, including the following sources of information: Medical Literature Analysis and Retrieval System Online (MEDLINE), through the US National Library of Medicine National Institutes of Health (PubMed) search engine; Latin American and Caribbean Literature in Health Sciences (LILACS), through the Virtual Health Library (VHL); Cumulative Index to Nursing and Allied Health Literature (CINAHL); Web of Science.

Descriptors in Health Sciences (DeCS) and Medical Subject Headings (MeSH) (baths, Infant, Newborn, Infant, Premature, Intensive Care Units, Neonatal) were used. associated by the Boolean AND operator and their respective synonyms by the OR operator. The strategy was standardized in MEDLINE/PubMed, and reproduced in other data sources according to the specific criteria of each one: “Infant, Newborn”[Mesh] OR (Infants, Newborn) OR (Newborn Infant) OR (Newborn Infants) OR (Newborns) OR (Newborn) OR (Neonate) OR (Neonates)) AND (“Intensive Care Units, Neonatal”[Mesh] OR (Newborn Intensive Care Unit) OR (Neonatal Intensive Care Unit) OR (Newborn Intensive Care Units (NICU)) OR (Neonatal ICU Newborn ICU) OR (ICU, Newborn) OR (ICUs, Newborn) OR (Newborn ICUs) OR (Newborn Intensive Care Units) OR (Neonatal Intensive Care Units) OR (ICU, Neonatal) OR (ICUs, Neonatal) OR (Neonatal ICUs). A total of 15 studies published between 2015 and 2022 were included, which addressed care with bathing NB in bed. Data were exported to an Excel® spreadsheet and refined for the construction of script scenes/steps’ content.

The scripts of the two videos, constructed from bibliographic survey, considered six scenes to present the technique of bathing NB’s bed in a neonatal unit, namely: 1 - Approaching parents and/or family members; 2 - Environment and material organization; 3 - Preparing newborns; 4 - Bath; 5 - After-bath care; and 6 - Environment and nursing note organization. The storyboard for recording described, through freehand drawings made by the main researcher, the filming plans, and the plan for editing covered the texts, formatting, narration and background music.

After preparing the first versions of the script and storyboard, the content validity process began with specialists in the second phase and video recording: production. Participants were contacted via email, and at each stage of the validity process, an instrument was developed in HyperText Markup Language (HTML) on Google Forms, filled out via the web, in three parts: participant personal and professional identification; script, storyboard or edited video; and general analysis based on the mentioned instruments. The response option for the items was a Likert scale with five weights (“totally agree” and “agree”, grouped as agreement, and “totally disagree” and “disagree”, grouped as disagreement).

After recording, LIBRAS was included by an interpreter hired by the researchers, following the mandatory elements, in accordance with ABNT NBR 15290 norms^(12)^.

The scenario in the videos simulated Neonatal Intensive Care Unit (NICU) beds: one with an incubator and another heated crib, including a bedside table, multiparameter monitor and armchair. Before the official recording, rehearsals were carried out to go over the script’s and storyboard’s content with those involved and to verify the equipment and actor positioning. Adjustments were made to achieve the technique’s good quality, in addition to features such as 4K resolution and shot variations (medium, pin and close) for the same scene. The audio was recorded in a studio with acoustic insulation by one of the researchers. The recording equipment was two Sony A6500 cameras with 35 mm, 70-200 mm and 16 mm lenses, video tripod, led light and H6 zoom recorder with lapel.

After recording, the scenes were edited in the post-production phase. The editing program was Final Cut Pro X, and the creation of the intro animation and inclusion of moving texts was Adobe After Effects®. Recording and editing were conducted by the researchers followed by professionals with experience in neonatology and audiovisual technicians. The soundtrack that composed the video along with the narration was the instrumental “carefree” by public domain artist Kevin MacLeod. Then, the edited video was subjected to validity and appearance assessment by specialists and nursing students.

### Analysis of results, and statistics

Data were stored in a database in Excel® format extracted from Google forms. Then, they were imported into the Statistical Package for the Social Sciences (SPSS) program version 21.0, submitted to descriptive statistics for frequency and percentage analysis, position measurements (mean and median) and variability (standard deviation). Agreement among judges was analyzed using the Content Validity Index (CVI), considering the weights “totally agree” and “agree”, grouped as agreement, and “completely disagree” and “disagree”, grouped as disagreement. The formula used in the calculation was: CVI = agreement/total responses, with items with agreement above 0.80 being valid^(13)^.

Script, storyboard, and edited video reliability was analyzed by Cronbach’s alpha, which checks the internal consistency of a single multi-item construct. Values above 0.80 were considered of high reliability^(13)^. In the analysis of nursing students’ assessment, in addition to descriptive statistics, the Wilcoxon test was applied, with a 95% Confidence Interval for the proportion of maximum scores (equal to 5) using a binomial distribution. “Strongly disagree” was the minimum score (1 point), and “strongly agree” was the highest score (5 points)^(14)^. Suggestions for adjustments were incorporated and the instrument was forwarded to PhD nurses for further analysis, following the Delphi technique precepts.

## RESULTS

Two videos were developed that represented a bed bath in the neonatal unit, lasting seven minutes each, “Good practices: bathing newborns in the heated crib”^(15)^ and “Good practices: bathing newborns in the incubator”^(16)^, lasting seven minutes each. The videos covered nursing interventions and specific care from preparation to bathing, such as donning, checking water temperature with a thermometer, proper management of NB, winding up, handling in pairs and encouraging the participation of a family member: the mother.

The video “Good practices: bathing newborns in the heated crib” hypothetically presents a preterm NB, 35 weeks old, male, 11 days old, current weight 2,100 g, using an orogastric tube and a central catheter inserted peripherally in the limb top right. The video “Good practices: bathing newborns in the incubator” presents a preterm NB, 31 weeks old, female, 20 days old, current weight 1,400 g, using an orogastric tube, both with indication of bed bath by GA. Emphasis was placed on protecting the venous device with transparent plastic film and encouraging the post-bath kangaroo position for better thermal regulation.

**Figure 1:**
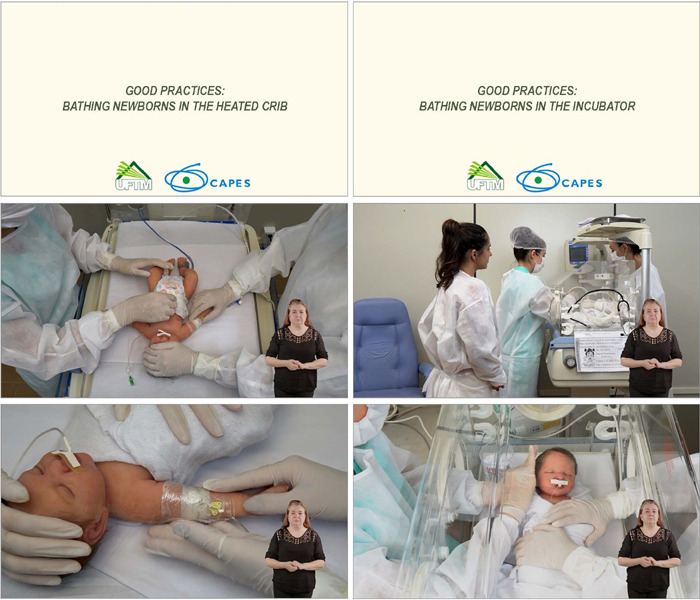
Images from the videos “Good practices: bathing newborns in the heated crib” and “Good practices: bathing newborns in the incubator”, Uberaba, Minas Gerais, Brazil, 2022

The final version of the videos after editing included introduction with title, institution and funding body logos, nine nursing interventions, the six scenes and the credits (Figure 1).

In terms of validity, among the 16 PhD nurse judges, 15 (93.7%) were female and one (6.3%) was male. In addition, 11 (68.7%) were from the state of Minas Gerais, two (12.4%) from São Paulo, one (6.3%) from Sergipe, one (6.3%) from Maranhão and one (6.3%) from Santa Catarina. Of these, ten (63.4%) worked in an undergraduate nursing course, three (18%) in Maternal and Child Units, two (12.4%) in teaching and research at a teaching hospital and one (6.2%) in a technical nursing course. Training time ranged from six to 35 years, with an average of 16.3 years.

The three specialists in social communication were male and from the state of Minas Gerais. Two (66.6%) worked with video recording and editing in an educational institution and one (33.4%) was a professor with a PhD.

Of the 43 nursing team members, all were female and from the state of Minas Gerais. In addition, 33 (76.7%) were nurses and ten (23.3%) were nursing technicians. All working in Maternal and Child Units, 30 (69.7%) in rooming-in and 13 (30.3%) in intensive care. Working time ranged from five to 20 years, with an average of 11.4 years.

The three specialists in LIBRAS were female and from the state of Minas Gerais. Furthermore, two (66.6%) worked with video recording and editing in an educational institution and one (33.4%) was a professor with a PhD.

In video assessment, carried out with 23 nursing students, 22 (95.7%) of them were female, with an average age of 24 years, minimum of 22 and maximum of 31 years. All were studying nursing at a public institution and, of these, 16 were in the tenth period, four in the new, one in the eighth and two in the seventh.

In the first round of script and storyboard content and appearance validity by PhD nurses, the indices were above 0.93 supporting the inclusion of all items for agreement and reliability, with minor reformulations according to the suggestions. These were: include parents in the scene for a humanized assistance, complete donning, not throw water directly on the NB’s skin because it is a great stimulus and change the bed after bathing NB when skin-to-skin contact is not possible. The suggestions were accepted and, after modifications, the script and storyboard were forwarded to PhD nurses for a second round, with no new suggestions. The assessed items indicated that the script and storyboard presented coherent objectives, clear content for understanding the theme with relevance to care practice, and environment and language appropriate to the context and target audience. The CVI and Cronbach’s alpha of the first and second rounds of this stage are presented in Table 1.

**Table 1:** Content Validity Index and Cronbach’s alpha of script and storyboard content validity and appearance, Uberaba, Minas Gerais, Brazil, 2022

Population	Judge nurses	
	Round 1 (n=16)	Round 2 (n=10)
Items	IVC	
Objectives		
Objectives are consistent with NB bath practice.	0.93	1.00
Objectives are consistent with the objectives proposed in the research.	0.96	0.98
Objectives are suitable to be carried out.	0.96	0.98
Content		
The content presented in the script corresponds to the objectives proposed in the work.	0.93	0.94
Content facilitates the teaching-learning process on the subject.	0.97	1.00
Content allows understanding of the topic.	0.97	1.00
Content follows a logical sequence.	0.97	0.96
Content incorporates all the necessary steps for carrying out NB bath.	0.97	1.00
Content has all the necessary materials for NB bath.	0.97	0.96
The information that the script presented is correct.	0.97	1.00
Relevance		
Images and scenes illustrate important aspects for NB bath practice.	0.95	1.00
Images and scenes are relevant for bathing NB to be of high quality.	0.97	1.00
Images and scenes allow transfer and generalization of learned content to different contexts.	0.97	1.00
Environment		
The setting is suitable for streaming the video.	0.96	0.98
The setting is suitable for learning the theme.	0.96	1.00
Verbal language		
Verbal language used in the script is accessible to the target audience.	0.95	1.00
Verbal language is easy to assimilate.	0.96	0.98
Inclusion of topics		
Educational video’s objective.	0.96	1.00
Purpose of NB bath.	0.97	1.00
Proper sequence and care before, during and after bathing.	0.97	1.00
Cronbach’s alpha	**0.99**	**0.99**

The indices of the first round of validity of video appearance by PhD nurses were above 0.98, attesting to the agreement and reliability of the included scenes. Relevant suggestions have been incorporated to improve audience understanding, such as adjusting text time to facilitate reading, inserting the list of necessary materials and sentence “change gloves to perform oral hygiene”.

After editing, the video was forwarded to PhD nurses for a second round. There were no new suggestions and the second version was sent to social communication specialists, specialist nurses and nursing students without further consideration. The assessment indicated that the video is configured as a teaching-learning tool about NB bed bath, easy to use, with adequate duration for the number of scenes, good lighting and clear narration. The CVI and Cronbach’s alpha of the rounds in this stage are shown in Table 2.

**Table 2:** Content Validity Index and Cronbach’s alpha of appearance validity of the edited video, Uberaba, Minas Gerais, Brazil, 2022

Items/participants	Judge nurses		Social communication specialists	Nursing team
	Round 1 n=(16)	Round 2 n=(16)	Round 1 n=(3)	Round 1 n=(43)
	CVI			
Functionality				
Video presents itself as an adequate tool for the purpose for which it is intended.	1.00	1.00	0.90	1.00
Video makes it possible to generate positive results in the teaching-learning process on the subject.	1.00	1.00	0.90	0.99
Usability				
Video is easy to use.	1.00	1.00	0.90	1.00
It is easy to learn the theoretical concepts used and their applications.	1.00	1.00	0.90	0.99
Allows users to easily apply the concepts worked on in hospital practice.	1.00	0.98	0.90	1.00
Efficiency				
Video duration (used time) is suitable for users to learn the content.	1.00	1.00	0.90	1.00
The number of scenes is consistent with the time proposed for the video.	1.00	1.00	0.95	1.00
Audiovisual technique				
Lighting is adequate for observation of practice.	1.00	1.00	0.90	1.00
The narrator’s tone and voice are clear and appropriate.	1.00	1.00	0.90	0.96
Video narration is used in an efficient and comprehensible way for the clientele.	1.00	1.00	0.95	1.00
It is possible to return to any part of the scenes when desired.	1.00	1.00	0.90	1.00
Environment				
Video reflects daily hospital practice.	1.00	1.00	0.90	1.00
The laboratory environment did not interfere with the fidelity of the bathing procedure in NB.	1.00	1.00	0.90	1.00
Procedures				
Educational video’s objectives.	1.00	0.98	0.90	0.97
Importance of bathing in NB and maternal bonding during bathing.	1.00	0.98	0.90	0.98
There was a complete presentation of the materials used in the procedure.	1.00	1.00	0.95	1.00
The steps of NB bath procedure are adequate and could be identified.	0.98	1.00	0.90	0.96
Cronbach’s alpha	**1.00**	**1.00**	**0.99**	**0.99**

The indices of LIBRAS validity items were above the recommended level (CVI=1.00), indicating that language was adequate to content and recommended norms. The items assessed by the three specialists in LIBRAS were: LIBRAS agrees with the audio narration; the interpreter is properly positioned on the screen; the interpreter window is well positioned and in focus; and it is possible to identify all the interpreter’s movements and gestures.

After the validity stage with specialists, the edited video was assessed by 23 nursing students using the same criteria in a single round. All domains had a mean score equal to 4.86±0.45 with a minimum of 3 and a maximum of 5 points, standard error of 0.95, median of 5.00 and p<0.001, indicating that the video was well assessed by students.

## DISCUSSION

Knowing that routine bathing in a neonatal care unit can cause changes in NB clinical stability due to excessive manipulation, exposure to low temperatures, variation in oxygen saturation and other factors, capable of negatively interfering with the safety of these highly vulnerable patients, it is important to invest in innovative teaching objects and tools that are attractive for nursing learning in this scenario^(1, 3)^.

In the video produced, the bath is performed by two people with cotton soaked in warm water and with the participation of mothers. For a premature baby, touch can be a harmful stimulus, so water should not be directly thrown on the NB’s skin^(1)^. Handling in pairs provides better postural organization for NB, because, while one professional performs care, the other maintains the alignment and containment of arms and legs, reducing stress due to agitation, excessive handling and procedure time. In this context, specific care was included in the video, such as a firm touch with the hands still on NB’s chest to lower the level of the incubator or heated crib and changing the diaper with NB on its side^(17)^. The winding of NB observed in the video has a positive effect on the vital parameters mentioned, on crying time and on the level of stress, pain and agitation, and is indicated in any type of bath^(4)^.

In the daily life of a NICU, family members present must be included in NB care to build a bond whenever possible^(18, 19)^. Physical contact with the NB through touch and the kangaroo position and being able to be present and participate in care, even while observing, provide feelings of closeness and trust and strengthen the bond among family members. Commitment to care contributes to the development of parenting during hospitalization, breaking down the barriers generated by the NICU’s complex and technological environment. Thus, nurses should encourage the presence and participation of family members in care^(1)^.

A study that developed and assessed an educational video on relief of acute pain in babies pointed out that the presence of family contributes to NB development and construction of bonds and affection among members and that using videos before procedures can bring them closer to content^(20)^.

The video developed in this study contemplates these precautions and visually demonstrates how to do them. This tool facilitates the teaching-learning process and contributes to acquisition of knowledge, as long as the methodological paths for its elaboration are respected, as a survey of the best scientific evidence on a given care and validity process by specialists, which allows assessing whether content is adequate for what is intended. In this regard, nursing has engaged in the production and validity of educational videos on various topics to be applied during continuing education, training and professional training actions^(21)^.

To instigate viewers, the video must be dynamic, have attractive images consistent with reality and be short in duration. Realism provokes feelings and emotions, and brings it closer to everyday practice, and the step-by-step details contribute to the development of skill^(22)^. Scene duration and dynamics influence the interest of those who watch long videos longer than eight to 12 minutes, dispersing attention^(23)^. Therefore, the videos developed in this study fit the recommendation.

In the video developed in this study, the scenes were filmed in several planes to attract attention, and the scenes were viewed from above convey the feeling of bathing, bringing viewers closer to the scene. A study that assessed videos for clinical teaching pointed out that quality influences viewers’ understanding^(24)^. Incorporating videos into the teaching practices of students and professionals makes it possible to explore various low and high complexity topics and disseminate them on free online platforms that maximize reach without territorial limits^(6)^.

The inclusion of LIBRAS is a differential resource in the video, which allows accessibility for teaching the deaf. This is a limitation cited by other studies. It may improve communication among professionals and deaf parents/relatives about this procedure. It is difficult to find health professionals and professors trained to work with this public in teaching and care practice, creating barriers in the transmission of knowledge, which is why audiovisual technologies with translations can facilitate^(23)^.

### Contributions to nursing and health

The videos developed are presented as contemporary learning tools, subsidizing neonatal safety with standardization of the technique that includes specific care to prevent hypothermia, reduce stress and excessive handling and encourage the presence and participation of parents. In nursing, they will be able to mediate students’ and professionals’ teaching-learning process. As they are attractive and dynamic, arouse the interest of those who watch them, favoring actions. We highlight the inclusion of LIBRAS to expand content accessibility. Also, by presenting the material and human resources necessary for a safe, scientifically based bath, the videos can help managers in planning available and necessary resources for actually providing assistance based on scientific evidence.

### Study limitations

The limitation of this study can be attributed to the representation of a specific culture of technique and humanization in care regarding bathing in a neonatal unit, which may differ in other countries. Moreover, the material and human resources presented in the videos may be restricted in some units.

## CONCLUSION

This study allowed the development and validity of educational videos about the NB bath in bed in neonatal units by PhD nurses specialist in social communication, nursing staff and nursing students. Two videos were developed that could contribute to academic training, permanent education and professional training in the field of neonatology. The scenes were divided into six precautions and LIBRAS was included, and all items assessed had CVI and Cronbach’s alpha above 0.90. It is recommended that future research be carried out to assess the effectiveness and applicability of the video during these training activities.

The videos can be watched online and contribute to health education processes about bathing NB in a neonatal unit and the path to development can provide subsidies for the creation of new videos. Therefore, videos’ ability to translate scientific evidence into practice in an applicable, playful and accessible way can create means for the translation of scientific knowledge with potential for the real improvement of nursing care practice.

## References

[1] Evidence-Based Medicine Group, Neonatologist Society, Chinese Medical Doctor Association (2021). Guidelines for neonatal skin management in the neonatal intensive care unit (2021). Zhongguo Dang Dai Er Ke Za Zhi.

[2] Kusari A, Han AM, Virgen CA, Matiz C, Rasmussen M, Friedlander SF (2019). Evidence-based skin care in preterm infants. Pediatr Dermatol.

[3] Dhamodaran M, Firth C, Webber MA, Clarke P (2021). Bathing babies: current practices in UK neonatal intensive care units. Arch Dis Child Fetal Neonatal Ed.

[4] Huang Y, Zhou L, Abdillah H, Hu B, Jiang Y (2022). Effects of swaddled and traditional tub bathing on stress and physiological parameters of preterm infants: A randomized clinical trial in China. J Pediatr Nurs.

[5] Sousa LB, Braga HFGM, Alencastro ASA, Silva MJN, Oliveira BSB, Santos LVF (2022). Effect of educational video on newborn care for the knowledge of pregnant and postpartum women and their families. Rev Bras Enferm.

[6] Alves MG, Batista DFG, Cordeiro ALPC, Silva MD, Canova JCM, Dalri MCB (2019). Production and validation of a video lesson on cardiopulmonary resuscitation. Rev Gaucha Enferm.

[7] Forbes H, Oprescu FI, Downer T, Phillips NM, McTier L, Lord B (2016). Use of videos to support teaching and learning of clinical skills in nursing education: a review. Nurse Educ Today.

[8] Fleming SE, Reynolds J, Wallace B (2009). Lights... camera... action! a guide for creating a DVD/video. Nurse Educ.

[9] Ferreira MVF, Godoy S, Góes FSN, Rossini FP, Andrade D (2015). Lights, camera and action in the implementation of central venous catheter dressing. Rev Latino-Am Enfermagem.

[10] Fehring RJ (1987). Methods to validate nursing diagnoses. Heart Lung [Internet].

[11] Hennink MM, Kaiser BN, Marconi VC (2017). Code saturation versus meaning saturation: how many interviews are enough?. Qual Health Res.

[12] Associação Brasileira de Normas Técnicas (ABNT) (2005). Norma Brasileira ABNT NBR 15290. Acessibilidade em comunicação na televisão [Internet].

[13] Polit DF, Beck CT (2019). Fundamentos de pesquisa em enfermagem: avaliação de evidências para a prática de enfermagem.

[14] Lopes JL, Baptista RCN, Domingues TAM, Ohl RIB, Barros ALBL (2020). Development and validation of a video on bed baths. Rev Latino-Am Enfermagem.

[15] Silva MPC (2023). Banho do recém-nascido na unidade neonatal: incubadora[Video] [Internet].

[16] Silva MPC (2023). Banho do recém-nascido na unidade neonatal: berço aquecido[Video] [Internet].

[17] Hatfield LA, Murphy N, Karp K, Polomano RC (2019). A Systematic review of behavioral and environmental interventions for procedural pain management in preterm infants. J Pediatr Nurs.

[18] Soltani N, Seyedrasooli A, Jabraeili M, Mousavi S (2022). The effect of maternal multisensory stimulations on bath stress in premature infants: a randomized controlled clinical trial. Infant Behav Dev.

[19] Kim AR, Kim SY, Yun JE (2020). Attachment and relationship-based interventions for families during neonatal intensive care hospitalization: a study protocol for a systematic review and meta-analysis. Syst Rev.

[20] Nazario AP, Lima VF, Fonseca LMM, Leite AM, Scochi CGS (2021). Development and evaluation of an educational video for families on the relief of acute pain in babies. Rev Gaucha Enferm.

[21] Campos DC, Silva LF, Reis AT, Góes FGB, Moraes JRMM, Aguiar RCB (2021). Development and validation of an educational video to prevent falls in hospitalized children. Texto Contexto Enferm.

[22] Araújo CC, Lihsieh M, Antunes TF, Vidal AP, Araújo BG, Menezes EG (2022). Validação de vídeo instrucional sobre banho de ofurô em recém-nascido pré-termo para enfermeiros. Esc Anna Nery.

[23] Caetano GM, Daniel ACQG, Costa BCP, Veia EV (2021). Elaboration and validation of an educational video on blood pressure measurement in screening programs. Texto Contexto Enferm.

[24] Krumm IR, Miles MC, Clay A, Carlos Ii WG, Adamson R (2022). Making effective educational videos for clinical teaching. Chest.

